# Response of Bean (*Vicia faba* L.) Plants to Low Sink Demand by Measuring the Gas Exchange Rates and Chlorophyll *a* Fluorescence Kinetics

**DOI:** 10.1371/journal.pone.0080770

**Published:** 2013-12-04

**Authors:** Bo-Fang Yan, Wei Duan, Guo-Tian Liu, Hong-Guo Xu, Li-Jun Wang, Shao-Hua Li

**Affiliations:** 1 Beijing Key Laboratory of Grape Science and Enology and Key Laboratory of Plant Resources, Institute of Botany, the Chinese Academy of Sciences, Beijing, People’s Republic of China; 2 University of Chinese Academy of Sciences, Beijing, People’s Republic of China; 3 Key Laboratory of Plant Germplasm Enhancement and Specialty Agriculture, Wuhan Botanical Garden, the Chinese Academy of Sciences, Wuhan, People’s Republic of China; US Naval Reseach Laboratory, United States of America

## Abstract

**Background:**

The decline of photosynthesis in plants under low sink demand is well known. Previous studies focused on the relationship between stomatal conductance (*g*_s_) and net photosynthetic rate (*P*_n_). These studies investigated the effect of changes in Photosystem II (PSII) function on the *P*_n_ decline under low sink demand. However, little is known about its effects on different limiting steps of electron transport chain in PSII under this condition.

**Methodology/Principal Finding:**

Two-month-old bean plants were processed by removing pods and flowers (low sink demand). On the 1^st^ day after low sink demand treatment, a decline of *P*_n_ was accompanied by a decrease in *g*_s_ and internal-to-ambient CO_2_ concentration ratio (*C*_i_/*C*_a_). From the 3^rd^ to 9^th^ day, *P*_n_ and *g*_s_ declined continuously while *C*_i_/*C*_a_ ratio remained stable in the treatment. Moreover, these values were lower than that of control. W_k_ (a parameter reflecting the damage to oxygen evolving complex of the donor side of PSII) values in the treatment were significantly higher than their corresponding control values. However, RC_QA_ (a parameter reflecting the number of active RCs per excited cross-section of PSII) values in the treatment were significantly lower than control from the 5^th^ day. From the 11^th^ to 21^st^ day, *P*_n_ and *g*_s_ of the treatment continued to decline and were lower than control. This was accompanied by a decrease of RC_QA_, and an increase of W_k_. Furthermore, the quantum yield parameters *φ*_Po_, *φ*_Eo_ and *ψ*_Eo_ in the treatment were lower than in control; however, *C*_i_/*C*_a_ values in the treatment gradually increased and were significantly higher than control on the 21^st^ day.

**Conclusions:**

Stomatal limitation during the early stage, whereas a combination of stomatal and non-stomatal limitation during the middle stage might be responsible for the reduction of *P*_n_ under low sink demand. Non-stomatal limitation during the late stages after the removal of the sink of roots and pods may also cause *P*_n_ reduction. The non-stomatal limitation was associated with the inhibition of PSII electron transport chain. Our data suggests that the donor side of PSII was the most sensitive to low sink demand followed by the reaction center of PSII. The acceptor side of PSII may be the least sensitive.

## Introduction

The hypothesis for the end-product inhibition of photosynthesis was proposed over a century ago [1]. According to this concept, owing to low sink demand, the accumulation of assimilates in source leaves may be responsible for the reduction of the photosynthetic rate. This reduction is accomplished by inhibiting the activities of the related metabolic enzymes as a direct feedback, which is also called feed forward [2], [3]. Several studies focused on investigating the photosynthetic response of plants to sink-source manipulation. These studies attempted to understand the mechanism of change in photosynthesis in many perennial woody species or annual herbaceous plants under low sink demand [4]–[12].

Studies on ‘accumulation of assimilates’ in source leaves showed some evidence supporting the hypothesis of end-product inhibition of photosynthesis [13], [14]. However, other studies, including our study on peaches and apples did not show a relationship between assimilate accumulation and the reduction of net photosynthetic rate (*P*_n_) under low sink demand [4], [6]–[8], [11], [15]. The mechanism by which low sink demand affects photosynthesis is still unclear.

In general, a decrease in *P*_n_ is accompanied by partial stomatal closure of source leaves under low sink demand [16]. Hence, down-regulated stomatal conductance (*g*_s_) was suggested as the initial response to low sink manipulation [4], [6]–[8], [11].The reduction of intercellular CO_2_ concentrations (*C*_i_) caused by lower *g*_s_ restricts the rate of gas exchange via stomata, resulting in stomatal limitation of photosynthesis [17]. Furthermore, the structure and function of photosystem II (PSII) is inhibited or damaged, leading to a non-stomatal limitation of photosynthesis [6], [18]. Therefore, stomatal limitation of photosynthesis takes place mostly under low PAR (photosynthetic active radiation) or during the early period while the non-stomatal limitations occur under high PAR (i.e. around noon) or during the late period under low sink demand [19].

Inhibition of PSII on the electron donor side causes a decrease in chlorophyll fluorescence, whereas inhibition on the electron acceptor side increases it [20], [21]. Thus, chlorophyll fluorescence may be used to detect changes in the photosynthetic apparatus *in vivo* [21]–[23]. Since the non-destructive measurements can be done with a high resolution of 10 µs, Strasser et al. developed a method for the analysis of increase in kinetics of fast fluorescence [24]–[27]. All oxygenic photosynthetic materials investigated so far have shown a polyphasic fluorescence rise consisting of a sequence of phases, denoted as O, J, I, and P (OJIP test), where O stands for the minimum fluorescence level, P is for the peak, and J and I are intermediate levels between O and P levels [22], [23], [28]. The OJIP-test is a powerful tool for the *in vivo* investigation of the structural property and photosynthetic activity of PSII. This includes the activity of donor side (i.e. oxygen evolving complex, OEC), reaction center (i.e. reaction center chlorophyll of PSII, P680) and acceptor side (i.e. pheophytin (Phe), plastoquinone (Q_A_ and Q_B_)), for light absorption, energy trapping, and electron transport [23]–[32]. Hence, Chlorophyll *a* fluorescence technique helps understand the mechanism of down-regulation of *P*_n_ after the sink demand is reduced. Low sink demand and non-stomatal limitation of *P*_n_ cause a decrease in the fraction of light energy used in electron transport [6]. However, the limiting steps (donor acceptor side, reaction center or acceptor side) of electron transport chain are not well understood. Moreover, the response process under low sink demand is dynamic. Previous studies mainly focused on the diurnal variations in photosynthesis during the early period, or a few days (at most one week) after source-sink manipulation [6]–[8], [11], [19]. Therefore, studying the mechanism of long term effects of low sink demand on *P*_n_ would provide new insights into this process.

In the present study, we examined the short and long term response of photosynthesis to low sink demand in bean (*Vicia faba* L.) plants. We used photosynthetic gas exchange and the OJIP-test technique to study the limiting steps of photosynthetic electron transport (including donor side, reaction center, and acceptor side of PSII). The objective of this study was to understand the underlying relationship between net photosynthetic rate and the electron transport chain of PSII under reduced sink demand in higher plants.

## Materials and Methods

### Plant Materials

The experiment was carried out during the month of October and November in 2012 at the Institute of Botany, Chinese Academy of Sciences, in Beijing, China. Seeds of ‘Daqingshan’ fava bean of uniform size were sown in plastic pots (15 cm diameter) containing plant peat moss and garden soil (1∶1, v/v). Routine commercial bean production irrigation and pest control methods were applied. The plants were grown in a greenhouse and cultivated as a single shoot under natural sunlight conditions at 30–60% relative humidity, a temperature of 18–32°C, and noon maximum photosynthetically active radiation (PAR) of about 1000 µmol photons m^−2^ s^−1^ in greenhouse.

### Treatments

Two-month-old bean plants of uniform size with eight mature compound leaves and five pods were topped (Oct. 17), and were divided into two groups. Six days later (beginning at 1800 h, Oct. 23), one group was subjected to low sink demand (LS) manipulation. This was done by girdling the base of the shoot (a horizontal 5-mm-wide band) with a razor blade in order to block the transport of assimilates from source leaves to the root while allowing water flow via xylem. In addition, pods and flowers were removed completely to minimize sink demand. For the control group, a longitudinal girdling of the same area as the horizontal girdling band was applied to the same part of the plants, in order to minimize the possible effect of physical injury [6], [11]. Five biological replicates were taken for all experiments.

### Measurement of Photosynthetic Gas Exchange Parameters

Terminal leaflets of the third fully expanded compound leaves were sampled from plants subject to a fixed PAR of 1100 µmol photons m^−2^s^−1^, a flow rate of 500 µmol s^−2^, and a 6 cm^2^ leaf area. These leaflet samples were used to measure gas exchange parameters including *P*_n_, *g*_s_, *C*_i_ and ambient CO_2_ concentration (*C*_a_) using a portable photosynthesis system Li-6400 (Li-Cor Inc., Lincoln, NE). The measurements of photosynthetic gas exchange were carried out between 0800 h and 1600 h on October 24, one day after initiating sink-source manipulation. The measurements were recorded only one time at 1300 h (photosynthetically active radiation at noon is usually the highest during a day) from the day 3 to 21 (the end of the experiment) at an interval of 2 to 5 days.

### Chlorophyll a Fluorescence Kinetics Transient Analysis (OJIP-test)

The OJIP-test parameters were measured according to Luo et al. on the same leaves for which gas exchange estimations were done [33]. The measurements were carried out by using a Handy-Plant Efficiency Analyzer (Hansatech Instruments, King’s Lynn, Norfolk, UK) on leaves after dark adaptation for more than 15 min [34], [35]. The transients were induced by red light of about 3000 µmol m^−2^ s^−1^ provided by an array of six light emitting diodes (peak wavelength, 650 nm). The fluorescence signals were recorded from 10 µs to 1 s. The data acquisition rate was 10 µs for the first 2 ms and 1 ms each thereafter. The fluorescence signal at 50 µs was considered to be the level; therefore it was a true *F*_o_. The following data from the original measurements were used: *F*_m_: maximal fluorescence intensity; *F*_k_ : fluorescence intensity at 300 µs [required for calculation of the initial slope (*M*_o_) of the relative variable fluorescence (V) kinetics and W_k_]; *F*_j_ : the fluorescence intensity at 2 ms (the J-step); *F*_i_ : the fluorescence intensity at 30 ms (I-step) [26], [36]. The derived parameters were as follows: the parameter W_k_ on the donor side of PSII was assumed to represents the damage to oxygen evolving complex (OEC), W_k_ = (*F*_k_−*F*_o_)/(*F*_j_−*F*_o_); the parameter RC_QA_ was assumed to represents the density of Q_A_-reducing reaction centers (RCs), calculated as the number of active PSII RCs per cross section (CS) at t = t_m_: RC_QA_ = RC/CS_m_ = *φ*_Po_×(*V*_j_/*M*_o_)×(ABS/CS_m_), here, ABS represents the total photon flux absorbed by the PSII antenna pigments. According to the energy flux theory proposed by Strasser et al., the total ABS is partially trapped by PSII RCs, and the fraction of ABS used for reduce Q_A_ is labeled as TR. However, the electron transport flux from Q_A_ to Q_B_ is labeled as ET [24]; then the yield indices or flux ratios can be derived. The parameter *φ*_Po_ can be considered as the maximum quantum yield of primary photochemistry, calculated as the ratio of TR/ABS at t = 0: *φ*_Po_ = TR_o_/ABS = 1−*F*_o_/*F*_m_; the parameter *ψ*_Eo_ was assumed to the probability that a trapped exciton moves an electron into the electron transport chain beyond Q_A_^−^, *ψ*_Eo_ = *ET*_o_/*TR*_o_ = (*F*_m_−*F*_j_)/(*F*_m_−*F*_o_); the parameter *φ*_Eo_ can be considered as the quantum yield of the electron transport flux from Q_A_ to Q_B_ (at t = 0), *φ*_Eo_ = *ET*_o_/ABS = (*F*_m_−*F*_j_)/*F*_m_. All these parameters are shown in Table S1.

### Statistical Analyses

Data were processed with SPSS 13.0 for Windows, and each value of the means and standard errors in the figures represents five replicates. Differences were considered significantly at a probability level of *P*<0.05 by a *t* - test.

## Results

### Gas Exchange Parameters

**Figure 1:**
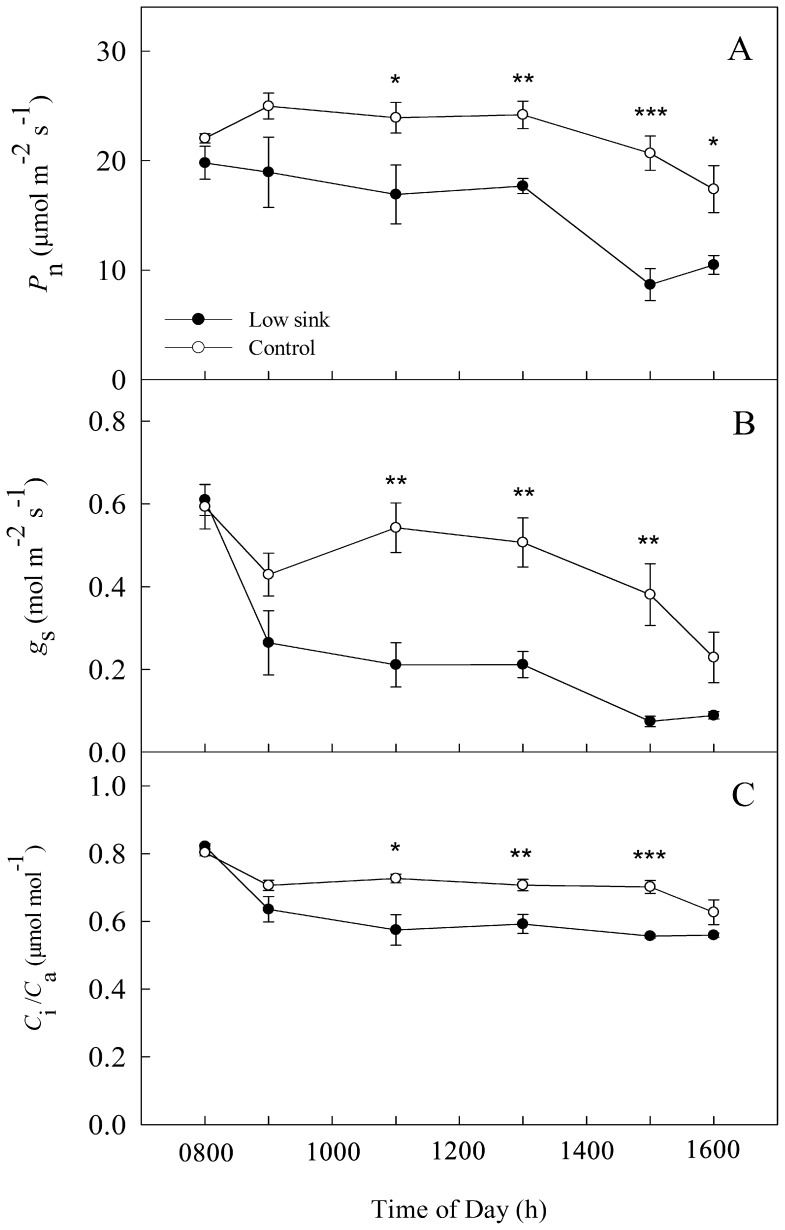
Diurnal variation in gas exchange parameters, including net photosynthesis rate (*P*_n_), stomatal conductance (*g*_s_) and internal-to-ambient CO_2_ concentration ratio (*C*_i_/*C*_a_) in bean source leaves in response to low sink demand on the 1^st^ day after removing the sink of roots and pods. Each value represents the mean of five replicates, and error bars represent ± S.E. The asterisks *, ** and *** indicate significant difference at *P*<0.05, 0.01 and 0.001 between the control and low sink, respectively.

For investigating the changes in gas exchange parameters under low sink conditions, we removed roots and pods of fava bean seedlings at 1800 h, Oct. 23 of 2012. At 0800 and 0900 h on the 1^st^ day after the low sink demand (LS) there were no differences on *P*_n_, *g*_s_ and *C*_i_/*C*_a_ (internal-to-ambient CO_2_ concentrations ratio) between LS and the control. However, *P*_n_, *g*_s_ and *C*_i_/*C*_a_ were significantly lower in the LS plants as compared with control from 1100 h to 1600 h (Fig. 1).

**Figure 2:**
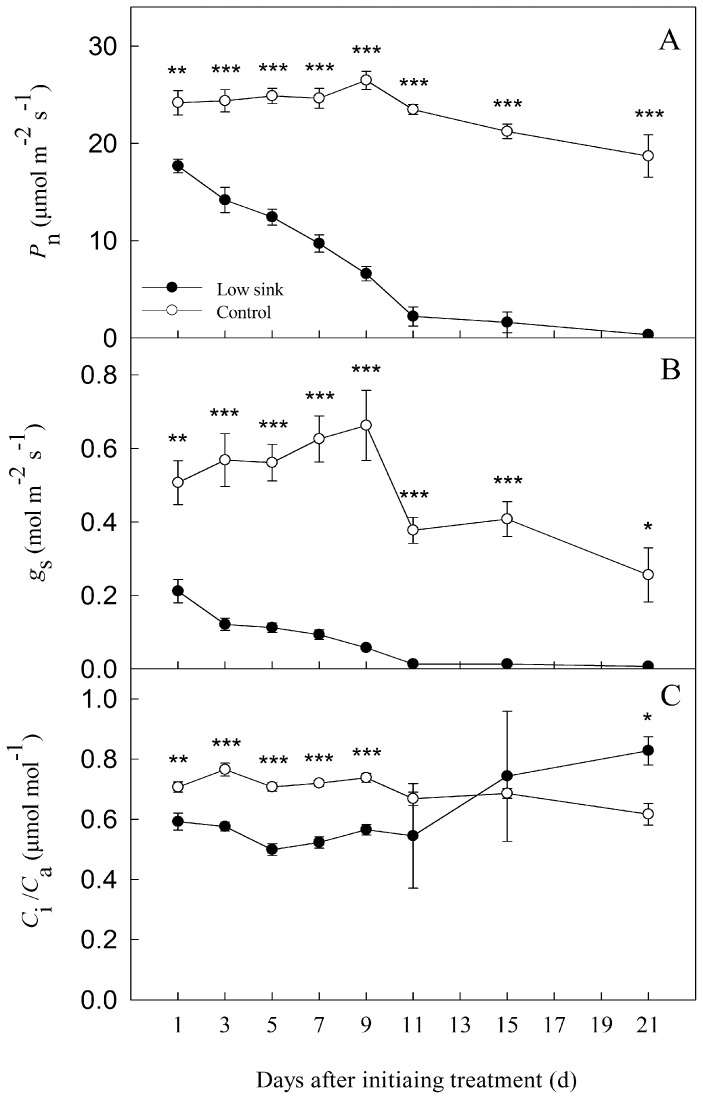
The response of gas exchange parameters including net photosynthesis rate (*P*_n_), stomatal conductance (*g*_s_) and internal-to-ambient CO_2_ concentration ratio (*C*_i_/*C*_a_) in bean source leaves to the treatment of low sink demand at 1300 h from the 1^st^ to 21^st^ day after removing the sink of roots and pods. Each value represents the mean of five replicates, and error bars represent ± S.E. The asterisks *, ** and *** indicate significant difference at *P*<0.05, 0.01 and 0.001 between the control and treatment, respectively.

As shown in Fig. 2, from the 3^rd^ to 9^th^ day after removing sink demand of roots and pods, *P*_n_, *g*_s_ and *C*_i_/*C*_a_ at 1300 h in LS plants were significantly lower than their controls. Moreover, *P*_n_ and *g*_s_ gradually decreased in the plants under low sink demand while they remained relatively stable in the control plants.

From the 11^th^ to 21^st^ day, *P*_n_ values at 1300 h in LS plants were still significantly lower than control, accompanied by lower *g*_s_. Moreover, *P*_n_ in the plants under low sink demand continuously declined, and *g*_s_ values were almost zero. During this period, *C*_i_/*C*_a_ in the LS plants gradually rose, and reached the highest value on 21^st^ day; moreover, this was higher than in the control (Fig. 2).

### Chlorophyll *a* Fluorescence Parameters

**Figure 3:**
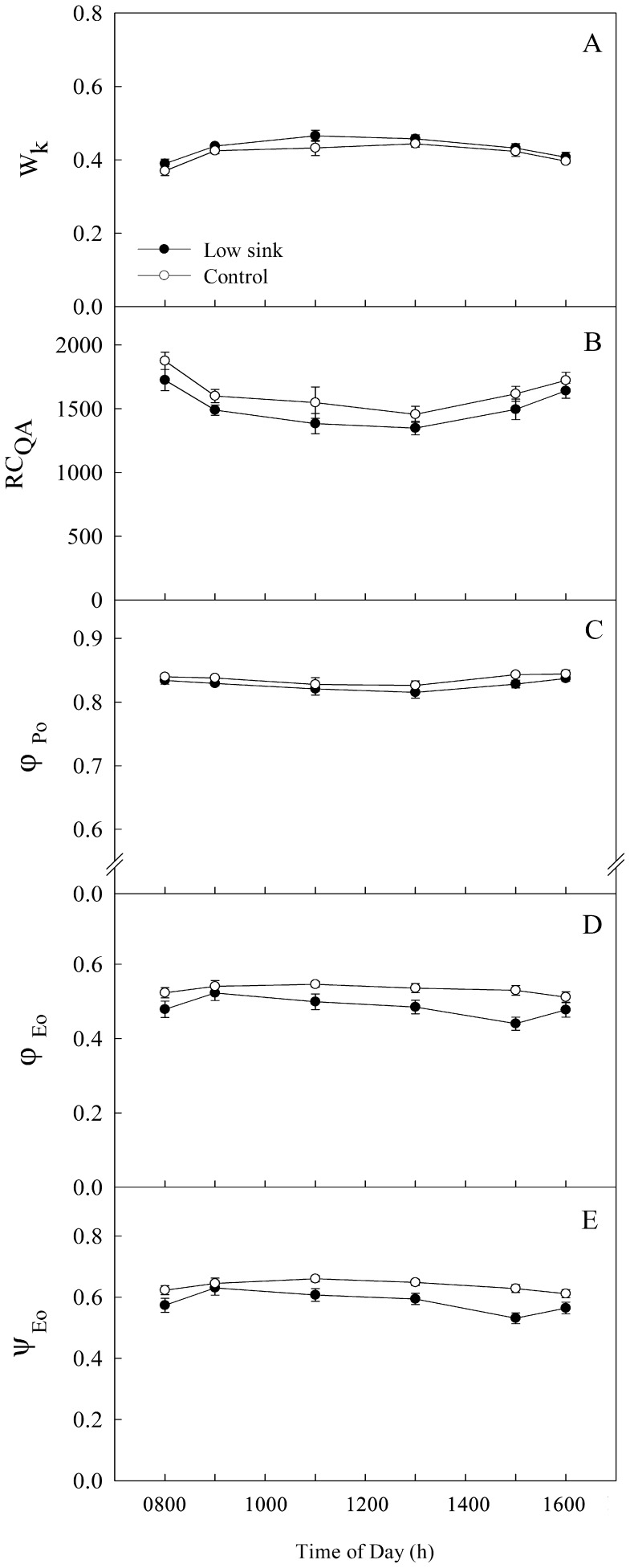
The change in parameters including W_k,_ RC_QA_, *φ*_Po_, *φ*_Eo_ and *ψ*_Eo_ involved in electron transport chain of PSII in bean (*V. faba* L.) source leaves on the 1^st^ day after removing the sink of roots and pods. Each value represents the mean of five replicates, and error bars represent ±S.E. The asterisks *, ** and *** indicate significant difference at *P*<0.05, 0.01 and 0.001 between the control and treatment, respectively.

OJIP test was conducted in order to explore the relationship between *P*_n_ decline and electron transport chain of PSII under LS treatment. We conducted the OJIP test at 0800 and 0900 h on the 1^st^ day after sink demand of roots and pods were removed. OJIP-test parameters W_k_, RC_QA_, *φ*_Po_, *φ*_Eo_ and *ψ*_Eo_ remained relatively stable throughout the day in both control and LS plants on the first day after the sink demand of roots and pods was removed. Moreover, there were no significant differences in these parameters between LS and the control (Fig. 3).

**Figure 4:**
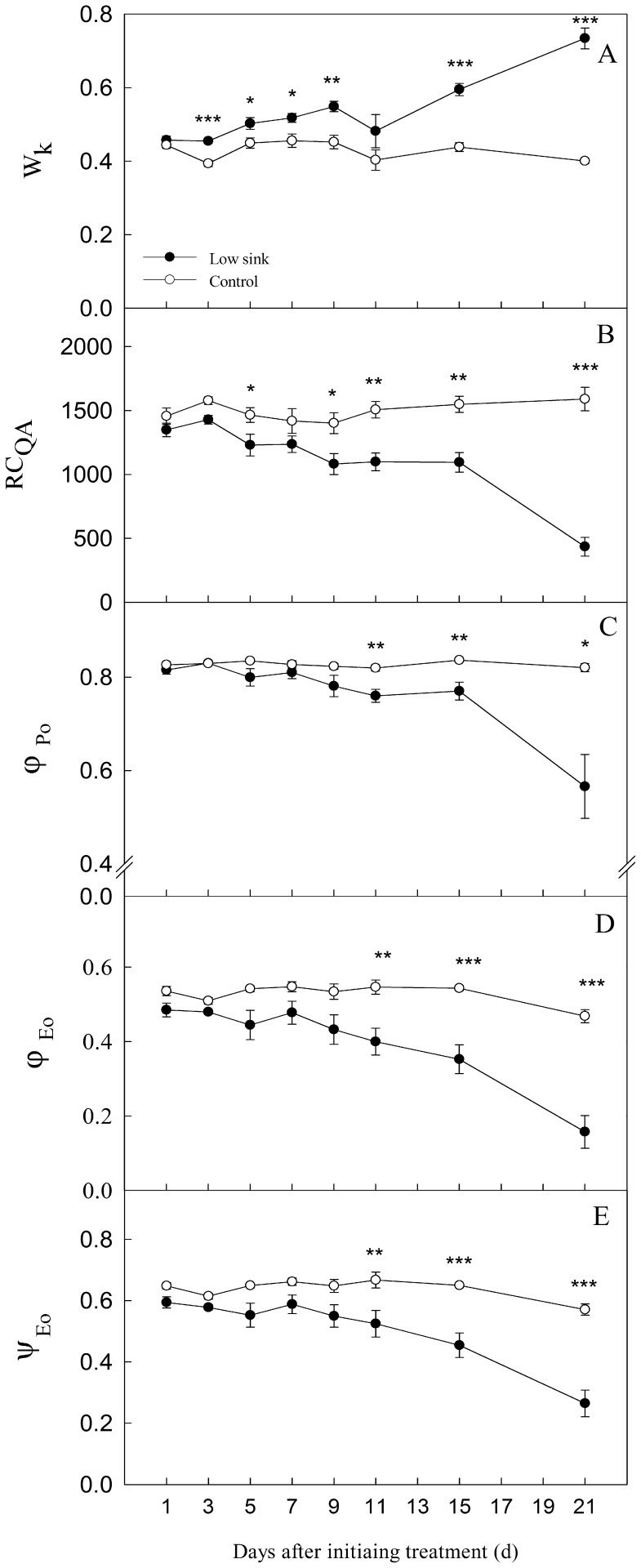
The change in parameters including W_k,_ RC_QA_, *φ*_Po_, *φ*_Eo_ and *ψ*_Eo_ involved in electron transport chain of PSII in bean (*V. faba* L.) source leaves at 1300 h from the 1^st^ to 21^st^ day after removing the sink of roots and pods. Each value represents the mean of five replicates, and error bars represent ± S.E. The asterisks *, ** and *** indicate significant difference at *P*<0.05, 0.01 and 0.001 between the control and treatment, respectively.

From the 3^rd^ day through the end of the experiment, W_k_, RC_QA_, *φ*_Po_, *φ*_Eo_ and *ψ*_Eo_ remained relatively stable in control plants. W_k_ increased, but RC_QA_, *φ*_Po_, *φ*_Eo_ and *ψ*_Eo_ decreased progressively in LS plants (Fig. 4). However, there were differences in the sensibility of these parameters in response to low sink demand of plants.

The W_k_ was sensitive to low sink demand in plants. From the 3^rd^ day after removing the sink of roots and pods, W_k_ values in LS plants were significantly higher than those in control (Fig. 4A). Moreover, the W_k_ of the last two measurements in LS plants on the 15^th^ and 21^st^ days after removing the sink demand increased sharply, resulting in about 35% and 83% higher values than control, respectively.

The RC_QA_ values in LS plants significantly differed from those in control from the 5^th^ day (Fig. 4B), then progressively decreased until the 15^th^ day after the sink was removed. A sharp decrease in RC_QA_ in LS plants was observed, but it was only 27% of the control value at the end of the experiment.

The parameters *φ*_Po_, *φ*_Eo_ and *ψ*_Eo_ of PSII showed a significant response to low sink was found. The treatment of low sink demand resulted in a significant decrease from the 11^th^ day (Fig. 4C, D, E). In LS plants, the values for *φ*_Po_, *φ*_Eo_ and *ψ*_Eo_ were about 69%, 33% and 46% of control values at the end of the experiment, respectively.

### Correlation of Different Parameters of Gas Exchange and Chlorophyll *a* Fluorescence under Low Sink Demand

Under low sink demand, *P*_n_ was positively correlated with *g*_s_, RC_QA_, *φ*_Po_, *φ*_Eo_ and *ψ*_Eo_ and negatively correlated with *C*_i_/*C*_a_ and W_k_ during the experimental period (Table 1). *g*_s_ was positively correlated with RC_QA_, *φ*_Po_, *φ*_Eo_ and *ψ*_Eo_ and negatively correlated with W_k_; however, *g*_s_ had no significant correlation with *C*_i_/*C*_a_. *C*_i_/*C*_a_ was positively correlated with W_k_ and negatively correlated with RC_QA_, *φ*_Po_, *φ*_Eo_ and *ψ*_Eo_. W_k_ was negatively correlated with RC_QA_, *φ*_Po_, *φ*_Eo_ and *ψ*_Eo_. There were positive correlations among RC_QA_, *φ*_Po_, *φ*_Eo_ and *ψ*_Eo_.

**Table 1:** Correlation among different parameters of gas exchange and chlorophyll *a* fluorescence under low sink demand.

	*P*_n_	*g*_s_	*C*_i_/*C*_a_	W_k_	RC_QA_	*φ*_Po_	*φ*_Eo_	*ψ*_Eo_
*P*_n_	1							
*g*_s_	0.944**	1						
*C*_i_/*C*_a_	−0.334**	−0.161	1					
W_k_	−0.717**	−0.609**	0.531**	1				
RC_QA_	0.721**	0.623**	−0.447**	−0.909**	1			
*φ*_Po_	0.610**	0.504**	−0.498**	−0.804**	0.862**	1		
*φ*_Eo_	0.652**	0.555**	−0.555**	−0.797**	0.821**	0.889**	1	
*ψ*_Eo_	0.613**	0.519**	−0.561**	−0.766**	0.757**	0.844**	0.989**	1

The asterisks **indicate a significant correlation at *P*<0.01.

## Discussion

The reduction of *P*_n_ in higher plants may result from stomatal or non-stomatal limitations or due to a combination of both [37]. When stomatal limitation of *P*_n_ occurs, *C_i_* (*C*_i_/*C*_a_) decreases in parallel with a decrease in *g*_s_. On the other hand, when non-stomatal limitation of *P*_n_ occurs, *C*_i_ (*C*_i_/*C*_a_) increases or remains stable in parallel with decreased *g*_s_ [37], [38]. Simultaneous measurement of chlorophyll *a* fluorescence extends this analysis, providing a means to determine the states of electron transport and energy partitioning in the photosynthesis apparatus including PSII and PSI [37], [39]. The *P*_n_ of plants under low sink demand declined significantly during the 1^st^ day after removing the sink of roots and pods. This was accompanied by a decrease in *g*_s_ and *C*_i_/*C*_a_ (Fig. 1). These results are similar to Setters’ results on the soybean plants [10]. Moreover, there were no significant differences in chlorophyll *a* parameters of PSII between LS and the control (Fig. 2). At this stage, there was a typical stomatal limitation of *P*_n_.

The chlorophyll *a* fluorescence transient analysis (OJIP-test) is a powerful tool in photosynthesis research to probe the PSII reactions, which may express the state of donor side, reaction center and acceptor side of PSII [31], [40], [41]. Any treatment or stress condition, which affects the donor side capacity will make the K-step apparent in the OJIP-test [31]. Therefore, the K-step can be used as an indicator of injury to the donor side. In general, the ratio W_k_ was used to show the changes in the amplitude in the K step; the higher the W_k_ values, the larger was the damage to the PSII donor side [31], [42]–[46]. In the OJIP-test, RC_QA_ is assumed to show the density of Q_A_-reducing PSII reaction center; thus lower the RC_QA_ values, the larger is the damage to PSII reaction center [41], [47]. Interestingly, from the 3^rd^ day to the 9^th^ day, as compared with control, *P*_n_ of the LS plants continued to decline, accompanied by a decrease of *g*_s_; however, *C*_i_/*C*_a_ remained relatively stable. Furthermore, during this period, W_k_ values in the LS plants were significantly higher while RC_QA_ values were lower than in control (Fig. 4). Therefore, *P*_n_ decline during this period was not mainly due to stomatal limitation, but also because of non-stomatal limitation. The change of W_k_ is the earliest among the OJIP parameters suggesting that the donor side might be the most sensitive to the treatment of low sink demand. Similarly, a decline in RC_QA_ under LS was observed from the 5^th^ day, indicating that the low sink demand may also result in the decrease in density of Q_A_-reducing PSII reaction centers during the middle period. In summary, the donor side of PSII was the first to be damaged, followed by damage to the reaction center.

From the 11^th^ to the 21^st^ day of LS treatment, *P*_n_ declined significantly, accompanied by a decrease in *g*_s_ and RC_QA_; however, there was an increase in W_k_ and *C*_i_/*C*_a_. In addition, other PSII parameters such as *φ*_Po_, *φ*_Eo_ and *ψ*_Eo_ in LS plants became lower than those in control. This was probably due to the damage to the acceptor side of PSII. The lower the *ψ*_Eo_ and *φ*_Eo_ values, the greater is the damage to PSII [48]–[52]. The acceptor side of PSII was much less influenced compared with the donor side and the reaction center under low sink demand. It can be speculated that when the acceptor side of PSII was damaged, the *P*_n_ decline was due to non-stomatal limitation. Moreover, throughout the LS treatment, *P*_n_ was positively correlated with *g*_s_, RC_QA_, *φ*_Po_, *φ*_Eo_ and *ψ*_Eo_ while it was negatively correlated with *C*_i_/*C*_a_ and W_k_ (Table 1). This suggests that non-stomatal limitation may contribute to the *P*_n_ reduction. Although previous results from peach trees [6], young apple trees [7], [53] and dahlia [11] showed that the decline in *P*_n_ in the late stages of low sink demand were because of non-stomatal limitation, these reports did not analyze the components of the PSII electron transport chain.

Interestingly, the increase in *C*_i_/*C*_a_ during the 15^th^ to the 21^st^ day after LS treatment is dramatically different from other parameters (Fig. 2). This increase is attributed to changes in ABA levels [37], starch accumulation [54], and leaf temperature [18], [55], etc. These factors probably interact with each other, or one of them may play a main role, or have different roles during different periods after the removal of sink from the plants.

## Conclusions

The reduction in *P*_n_ in source leaves of bean plants under low sink demand is not only caused by stomatal limitation during the early stages, but also by non-stomatal limitation during the middle and later stages after the removal of the sink of roots and pods. This inhibition of *P*_n_ decrease by non-stomatal limitation under low sink demand was associated with the inhibition of the PSII electron transport chain. Our results suggest that the donor side of PSII is the most sensitive to low sink demand, followed by the reaction center of PSII. The acceptor side of PSII was found to be the least sensitive to low sink demand. The reduction of the acceptor side ability of PSII occurred during the late period.

## Supporting Information

Table S1 **Summary of parameters, formulae and their description using data extracted from chlorophyll a fluorescence transient (OJIP-test).**
(DOCX)
