# Supplementary material for: Response of Bean (Vicia faba L.) Plants to Low Sink Demand by Measuring the Gas Exchange Rates and Chlorophyll a Fluorescence Kinetics
Source: PLoS One. 2013 Dec 4;8(12):e80770. doi: 10.1371/journal.pone.0080770 (PMC3851463; doi:10.1371/journal.pone.0080770)
Supplement: Table S1 — Summary of parameters, formulae and their description using data extracted from chlorophyll a fluorescence transient (OJIP-test). (DOCX) [file pone.0080770.s001.docx]

**Table S1** Summary of parameters, formulae and their description using data extracted from chlorophyll *a* fluorescence transient (OJIP-test)

| Fluorescence parameters | Fluorescence parameters Description |
| --- | --- |
| Extracted parameters |  |
| *F*_t_ | Fluorescence intensity at time ‘t’ after onset of actinic illumination |
| *F*_50 µs_ | Minimum reliable recorded fluorescence at 50 µs with the Handy PEA |
| *F*_k_ (F_300 µs_) | Fluorescence intensity at 300 µs |
| *F*_P_ | Maximum recorded (= maximum possible) fluorescence at P-step |
| Area | Total complementary area between fluorescence induction curve and *F* = *F*_m_ |
| Derived parameters |  |
| *F*_o_ ≌ *F*_50 µs_ | Minimum fluorescence, when all PSII RCs are open |
| *F*_m_ = *F*_P_ | Maximum fluorescence, when all PSII RCs are closed |
| *V*_j_ = (*F*_2 ms_ - *F*_o_)/(*F*_m_ - *F*_o_) | Relative variable fluorescence at the J-step (2 ms) |
| *V*_i_ = (*F*_30 ms_ - *F*_o_)/(*F*_m_ - *F*_o_) | Relative variable fluorescence at the I-step (30 ms) |
| W_K_ =( *F*_k_ - *F*_o_ )/( *F*_j_ - *F*_o_) | Represent the damage to oxygen evolving complex (OEC) |
| *M*_o_ = 4 (*F*_300 µs_ - *F*_o_)/(*F*_m_ - *F*_o_) | Approximated initial increment (in ms^-1^) of the relative variable fluorescence |
| *φ*_Po_ = TR_o_/ABS = 1 - (*F*_o_/*F*_m_) = *F*_v_/*F*_m_ | Maximum quantum yield of primary photochemistry at t = 0 |
| *φ*_Eo_ = ET_o_/ABS = (*F*_v_/*F*_m_) × (1 – *V*_j_) | Quantum yield for electron transport at t = 0 |
| *ψ*_Eo_ = ET_o_/TR_o_ = 1 - *V*_j_ | Probability (at time 0) that a trapped exciton moves an electron into the electron transport chain beyond Q_A_^-^ |
| RC_QA_ =*φ*_Po_ × (ABS/CS_m_) × (*V*_j_/*M*_o_) | Amount of active PSII RCs (Q_A_-reducing PSII reaction centers) per CS at t = m |
